# Integrated analysis of the salivary microbiome and metabolome in chronic and aggressive periodontitis: A pilot study

**DOI:** 10.3389/fmicb.2022.959416

**Published:** 2022-09-26

**Authors:** Yiping Wei, Meng Shi, Yong Nie, Cui Wang, Fei Sun, Wenting Jiang, Wenjie Hu, Xiaolei Wu

**Affiliations:** ^1^Department of Periodontology, National Engineering Laboratory for Digital and Material Technology of Stomatology, NHC Research Center of Engineering and Technology for Computerized Dentistry, National Clinical Research Center for Oral Diseases, Peking University School and Hospital of Stomatology, Beijing, China; ^2^Beijing Tiantan Hospital, Capital Medical University, Beijing, China; ^3^Laboratory of Environmental Microbiology, Department of Energy and Resources Engineering, College of Engineering, Peking University, Beijing, China

**Keywords:** periodontitis, salivary microbiome, metabolome, high-throughput nucleotide sequencing, gas chromatography-mass spectrometry

## Abstract

This pilot study was designed to identify the salivary microbial community and metabolic characteristics in patients with generalized periodontitis. A total of 36 saliva samples were collected from 13 patients with aggressive periodontitis (AgP), 13 patients with chronic periodontitis (ChP), and 10 subjects with periodontal health (PH). The microbiome was evaluated using 16S rRNA gene high-throughput sequencing, and the metabolome was accessed using gas chromatography-mass spectrometry. The correlation between microbiomes and metabolomics was analyzed by Spearman’s correlation method. Our results revealed that the salivary microbial community and metabolite composition differed significantly between patients with periodontitis and healthy controls. Striking differences were found in the composition of salivary metabolites between AgP and ChP. The genera *Treponema*, *Peptococcus*, *Catonella*, *Desulfobulbus*, *Peptostreptococcaceae_[XI]* (*[G-2]*, *[G-3] [G-4]*, *[G-6]*, and *[G-9]*), *Bacteroidetes_[G-5]*, *TM7_[G-5]*, *Dialister*, *Eikenella*, *Fretibacterium*, and *Filifactor* were present in higher levels in patients with periodontitis than in the healthy participants. The biochemical pathways that were significantly different between ChP and AgP included pyrimidine metabolism; alanine, aspartate, and glutamate metabolism; beta-alanine metabolism; citrate cycle; and arginine and proline metabolism. The differential metabolites between ChP and AgP groups, such as urea, beta-alanine, 3-aminoisobutyric acid, and thymine, showed the most significant correlations with the genera. These differential microorganisms and metabolites may be used as potential biomarkers to monitor the occurrence and development of periodontitis through the utilization of non-invasive and convenient saliva samples. This study reveals the integration of salivary microbial data and metabolomic data, which provides a foundation to further explore the potential mechanism of periodontitis.

## Introduction

Periodontitis, which causes alveolar bone resorption, is inflammation of periodontal tissue that results from the host immune response to bacterial infection (Marchesan et al., 2020). Periodontitis is the most common cause of tooth loss and can subsequently impair the quality of life (Petersen, 2003). Aggressive periodontitis (AgP) refers to rapidly progressing periodontitis that usually occurs in younger patients and differs from chronic periodontitis (ChP) according to the 1999 Classification of Periodontal Diseases and Condition (Armitage, 1999; Armitage, 2000). AgP constitutes a disease that causes considerable morbidity because of its pronounced destruction, which may lead to edentulism early in life. Some studies reported that AgP differs from ChP due to the age of the onset of the disease, rapid progression, the severity of disease, structure of subgingival flora, differences in host response, changes in response to treatment, and familial transmission characteristics of the disease (Emingil et al., 2007; Armitage and Cullinan, 2010).

Saliva is particularly promising as it contains locally produced proteins, as well as other components from the systemic circulation (Buduneli and Kinane, 2011). Moreover, saliva samples can be collected in an easy, non-invasive, repeated, minimal time-consuming manner. Salivary diagnostics has proved to be a promising substrate for the early detection of oral and systemic diseases (Buzalaf et al., 2020; Martina et al., 2020; Hyvarinen et al., 2021; Khurshid et al., 2021). Even so, various challenges persist regarding the use of saliva as a potential diagnostic media for periodontitis, mainly because of the lack of specific markers of the disease.

The microorganisms are believed to be involved in the pathogenesis of periodontitis. Moreover, it is now clear that some important factors that connect microbiota to periodontitis are microbial metabolites (Lu et al., 2013; Duran-Pinedo and Frias-Lopez, 2015). A few studies have used next-generation sequencing technologies to characterize the salivary microbiota in patients with AgP or ChP (Damgaard et al., 2019; Lundmark et al., 2019). These results revealed distinct and disease-specific patterns of salivary microbial composition between patients with periodontitis and healthy controls. Furthermore, several interventional studies have demonstrated the impact of non-surgical periodontal treatment on the composition of the salivary microbiome (Belstrom et al., 2018; Chen et al., 2018; Greenwood et al., 2020). It was reported that the salivary levels of periopathogens might be used as a biomarker of periodontitis (Yoshizawa et al., 2013; Kageyama et al., 2017).

The salivary metabolome is a rapidly evolving discipline aiming to reflect the real-time molecular phenotype of oral health (Liebsch et al., 2019; Gardner et al., 2020). The metabolic profile of saliva can give valuable information on the complex pathogenic mechanisms of periodontitis (Rzeznik et al., 2017; Romano et al., 2018; Kim et al., 2021). Several studies have shown its potential to reflect periodontal inflammation severity (Kuboniwa et al., 2016; Sakanaka et al., 2017). Also, non-surgical periodontal therapy may produce significant changes in the metabolic profile (Romano et al., 2019; Citterio et al., 2020).

Although increasing evidence is emerging on the variation in the salivary microbiota and metabolic characteristics of periodontitis, respectively, the relationship between host bacterial interactions and biochemical metabolism has not been identified. Thus, this pilot study was designed to identify the salivary microbial community and metabolic profiles in patients with generalized AgP and ChP, and subjects with periodontal health (PH) using 16S rRNA gene high-throughput sequencing and gas chromatography-mass spectrometry (GC-MS). The integration of microbiome and metabolomics can effectively provide a new sight regarding the influence of periodontitis.

## Materials and methods

### Study population and clinical examination

Ethics approval for this study was granted by the Ethics Committee of the Peking University Health Science Center (approval number: PKUSSIRB-201631135). All participants were individually informed and signed informed consent forms in accordance with the Declaration of Helsinki.

From January 2017 to August 2017, 13 patients with AgP and 13 patients with ChP were recruited from Peking University School and Hospital of Stomatology, and 10 PH subjects were selected as controls. The clinical inclusion criteria for PH and the diagnostic criteria for ChP and AgP have been previously described in detail (Shi et al., 2018). Exclusion criteria included systemic diseases, use of antibiotics or any other drugs that were known to affect periodontal conditions within the past 3 months, periodontal treatment in the past 6 months, a remaining tooth with caries, pregnancy or lactation, and smoking.

Full-mouth periodontal indices, including probing depth (PD), attachment loss (AL), and bleeding index (BI) (Mazza et al., 1981), were recorded at six sites per tooth by one practitioner (WH). His reliability was calibrated as described by Shi et al. (2018). All patients fulfilled the categorization of generalized Stage III or IV, Grade C periodontitis, according to the new periodontitis classification system (Tonetti et al., 2018).

### Sample collection

One week after the full-mouth periodontal examination, saliva samples were collected in the morning (around 8 a.m. to 9 a.m.). All subjects were restrained from food for 8 h and oral hygiene for 12 h before sampling. Each subject was instructed to allow saliva to be collected in the mouth, and let the saliva drain into a sterile graduated tube for 10 min. About 3 ml of unstimulated whole saliva was collected. After centrifugation, the saliva was immediately frozen at –80°C.

### Microbial DNA extraction and 16S rRNA gene sequencing

The bacterial DNA of saliva samples was extracted using the QIAamp DNA Mini Kit (QIAGEN Sciences, USA). Polymerase chain reaction amplification was performed using specific primers, 338F (5′, ACTCCTACGGGAGGCAGCAG, 3′) and 806R (5′, GGACTACNNGGGTATCTAAT, 3′), targeting the V4–V5 region of the bacterial 16S rRNA gene. Thermal cycling conditions were as follows: initial denaturation at 95°C for 3 min, followed by 24 cycles of denaturation at 95°C for 30 s, annealing at 56°C for 30 s, elongation at 72°C for 45 s, and finally at 72°C for 10 min. Purified amplicons were pooled in equimolar and paired-end sequenced using the Illumina MiSeq PE300 platform (Illumina, San Diego, USA). The datasets generated and analyzed during the current study are available from the NCBI Sequence Read Archive database (Accession Number: SRP173111; SRP228020).

### Gas chromatography-mass spectrometry metabolomics processing

Gas chromatography-mass spectrometry (GC-MS) was performed as described in a previous study (Shi et al., 2020). Briefly, 300 μL of methanol was used for extraction, following the addition of 20 μL of 2-chloro-l-phenylalanine (0.3 mg/mL) as an internal standard. All samples were analyzed with an Agilent Gas Chromatography Mass Spectrometer (7890A/5975C GC-MS System, Agilent, CA, USA). The GC system employed a chromatographic column (HP-5MS) (30 m × 0.25 mm × 0.25 μm, Agilent J & W Scientific, Folsom, CA, USA).

### Bioinformatic analysis, statistical analysis, and visualization

The sequences were analyzed using the Quantitative Insights Into Microbial Ecology (QIIME) software package, and alpha and beta diversity values were determined. After the OTU table was rarified, alpha diversity and beta diversity calculations were performed. Analysis of similarity (ANOSIM), permutational multivariate analysis of variance (Adonis), and multi-response permutation procedure (MRPP) were used to examine the differences in the microbial community structure between the groups. The composition and abundance depicted in the taxonomy profile were visualized by GraphPad PRISM^®^ software (version 4.0). Normality tests for each group of data were conducted. The Kruskal–Wallis test, Mann–Whitney test, and Student’s *t*-tests were performed using SPSS 20.0.

The raw data obtained in .D format from the platform were converted to .CDF format by using ChemStation analysis software (version E.02.02.1431, Agilent, CA, USA), and then imported into Chroma TOF software (version 4.34, LECO, St Joseph) for preprocessing. The remaining steps were carried out as described in the literature (Shi et al., 2020). Unsupervised principal component analysis (PCA) and (orthogonal) partial least-squares analysis [(O) PLS-DA] were then applied to determine the differences in metabolic profiles between the groups. In the (O)PLS-DA analysis, variable importance in the projection (VIP) value greater than 1 and *p-*values (two-tailed Student’s *t*-test) less than 0.05 were considered to be different variables. Metabolites were then identified by searching in a self-built database of the Majorbio I-Sanger Cloud Platform,^1^ which integrated the data of the Human Metabolome Database, KEGG Compound Database, and Lipid Maps Structure Database.

The correlation between microbiomes and metabolomics was analyzed by the Spearman method. A heat map of Spearman’s rank correlation coefficient was used to illustrate the relationship between microbial communities and metabolites.

## Results

### Microbial signatures of saliva samples

The demographic characteristics and clinical parameters are presented in Table 1. A total of 36 subjects, including 13 patients with AgP, 13 patients with ChP, and 10 PH, were included in this study. PD, AL, and BI values were significantly higher in the group with periodontitis (AgP and ChP) than in the PH subjects (*p* < 0.05). The mean age in the ChP group was significantly higher than in the other two groups.

**TABLE 1:** Demographic and clinical parameters of the study subjects.

	AgP (*n* = 13)	ChP (*n* = 13)	H (*n* = 10)
Age	29.4 ± 4.2^c^	38.2 ± 5.9^bc^	26.5 ± 1.7^b^
Gender			
Male	4	7	4
Female	9	6	6
Full mouth			
PD (mm)	5.0 ± 1.1^a^	4.3 ± 0.7^b^	2.4 ± 0.2^ab^
number of sites with PD > 5mm	33.5 ± 8.2^a^	31.7 ± 4.1^b^	0**^ab^**
BI	3.8 ± 0.3^a^	3.7 ± 0.8^b^	0.4 ± 0.3^ab^
BOP (+)%	98.6 ± 0.4^a^	99.2 ± 0.5^b^	15.0 ± 0.4^ab^
AL (mm)	4.8 ± 1.0^a^	4.0 ± 0.4^b^	0^ab^

Values are means ± standard deviations. PD, probing depth; BI, bleeding index; BOP, bleeding on probing; AL, attachment loss. ^a^Indicate statistically significant differences between AgP and PH, ^b^indicate statistically significant differences between ChP and PH, ^c^indicate statistically significant differences between AgP and ChP.

Sequences were clustered to 888 OTUs based on 97% sequence similarity. A total of 12 phyla, 30 classes, 54 orders, 92 families, and 173 genera were detected. The rarefaction curves suggested that the sequencing depth was adequate (Supplementary Figure 1).

**FIGURE 1:**
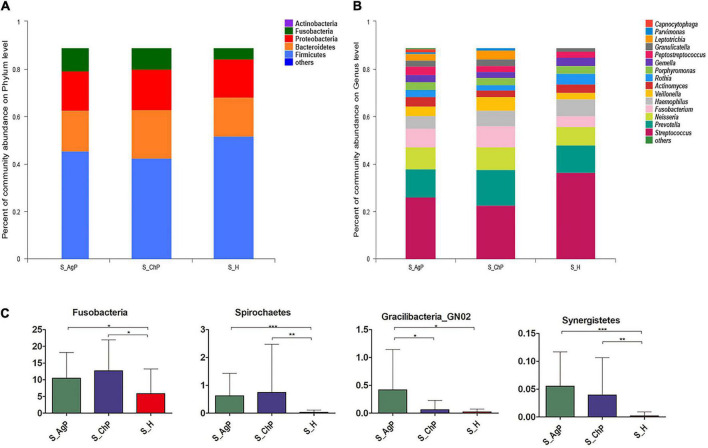
Bacterial composition of three groups. Microbial community composition at the phylum level **(A)** and genus level **(B)** of salivary samples in the aggressive periodontitis group (shown as S_AgP), chronic periodontitis group (shown as S_ChP), and healthy group (shown as S_PH). The relative abundance of bacterial taxa at the phylum level shows significant differences among groups **(C)**. *p < 0.05; **p < 0.01; ***p < 0.001.

In the AgP, ChP, and PH groups, the five most abundant phyla were Firmicutes, Bacteroidetes, Proteobacteria, Fusobacteria, and Actinobacteria, which represented more than 97% of the total sequences (Figure 1A). At the genus level, *Streptococcus, Prevotella, Neisseria, Fusobacterium, Haemophilus, Veillonella, Actinomyces, Rothia, Porphyromonas, Gemella, Peptostreptococcus, Granulicatella, Leptotrichia, Parvimonas*, and *Capnocytophaga* accounted for more than 85% of the sequencing results in all saliva samples, though there were some differences in the precise proportions among the AgP, ChP, and PH groups (Figure 1B).

Then we compared the relative abundance of microbiota between samples from periodontitis and healthy subjects. The abundance of Fusobacteria, Spirochetes, and Synergistetes increased significantly in the periodontitis group. The abundance of Gracilibacteria_GN02 was significantly higher in AgP than in ChP and PH (Figure 1C). At the genus level, 43, 31, and 5 genera were distributed differently between AgP vs. PH, ChP vs. PH, and AgP vs. ChP, respectively (Supplementary Tables 1–3). Most significant differences were observed in the low-abundance genera. *Leptotrichia*, *Treponema*, *Peptococcus*, *Catonella*, *Desulfobulbus*, *Peptostreptococcaceae_[XI] ([G-2]*, *[G-3] [G-4]*, *[G-6]*, and *[G-9])*, *Bacteroidetes_[G-5]*, *TM7_[G-5]*, *Dialister*, *Eikenella*, *Filifactor*, *Fretibacterium*, *Lachnospiraceae_[G-8]*, *Mollicutes_[G-1]*, and *Slackia* increased significantly both in the ChP and AgP groups when compared to the PH group. *Agrobacterium, GN02_[G-2], GN02_[G-1], Mobiluncus*, and *Peptostreptococcaceae_[XI][G-6]* showed significant differences between the AgP and ChP groups.

**FIGURE 2:**
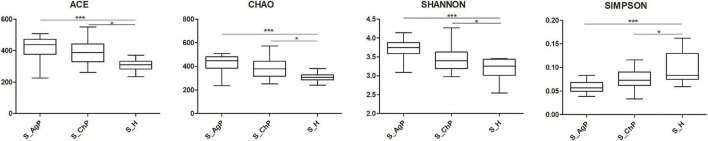
Alpha diversity indexes of microbial communities in three groups. The alpha diversity of microbiota as calculated by ACE index, Chao index, Shannon index, and Simpson index of saliva samples in aggressive periodontitis group (S_AgP), chronic periodontitis group (S_ChP), and healthy group (S_PH). The error bars indicate mean with standard error. *p < 0.05; ***p < 0.001.

**FIGURE 3:**
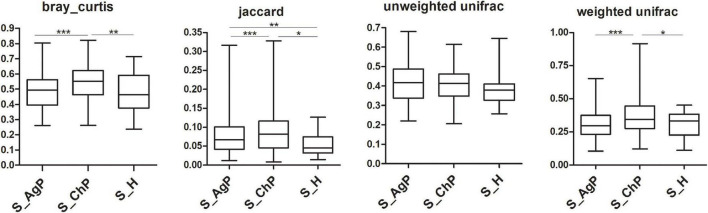
Beta diversity of saliva samples in three groups based on Bray-Curtis, Jaccard, weighted, and unweighted UniFrac distances. *p < 0.05; **p < 0.01; ***p < 0.001.

To illustrate the richness, evenness, and species diversity of the microbial community, α diversity indices, including the Chao index, ACE index, Shannon index, and Simpson index, were calculated. The results showed that the alpha diversity of subjects with periodontitis was greater than that observed in healthy individuals (Figure 2). The β diversity calculations based on Bray-Curtis, Jaccard, weighted, and unweighted UniFrac distances showed significant compositional shifts between periodontitis and healthy groups (Figure 3). ANOSIM, Adonis, and MRPP were used to further confirm that there was no significant difference between the AgP and ChP groups (Table 2).

**TABLE 2:** Differences in microbial community structure among the three groups (*p-*value).

	ANOSIM	Adonis	MRPP
S_AgP vs. S_H	0.033*	0.004**	0.001***
S_ChP vs. S_H	0.062	0.01**	0.015*
S_AgP vs. S_ChP	0.133	0.409	0.517

*p < 0.05, **p < 0.01, ***p < 0.001.

### Metabolic signatures of saliva samples

**FIGURE 4:**
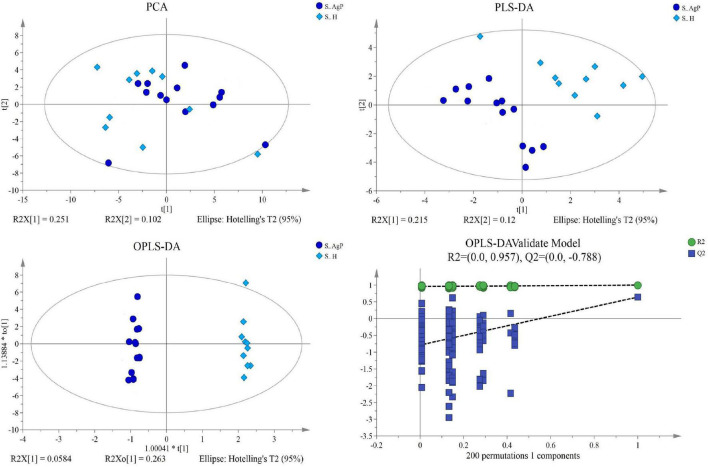
Dissimilarity in salivary metabolic signatures between AgP and PH. PCA analysis **(A)**, PLS-DA analysis **(B)**, OPLS-DA analysis **(C)**, and response permutation testing of OPLS-DA **(D)**. PCA, principal components analysis; PLS-DA, partial least-squares discriminant analysis; OPLS-DA, orthogonal partial least-squares discriminant analysis.

**FIGURE 5:**
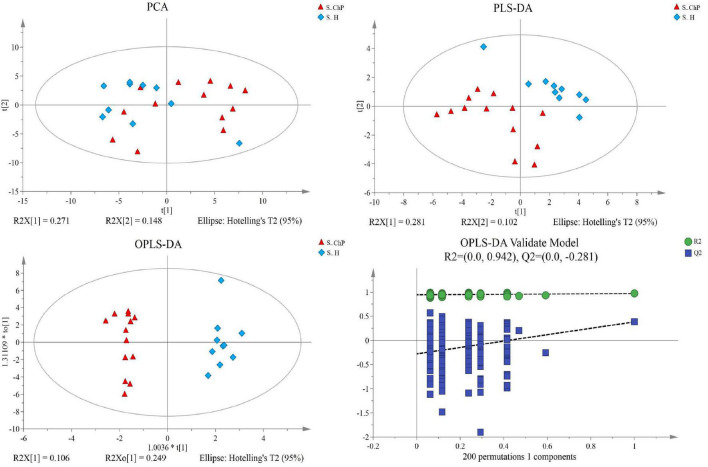
Dissimilarity of salivary metabolic signatures between ChP and PH. PCA analysis **(A)**, PLS-DA analysis **(B)**, OPLS-DA analysis **(C)**, and response permutation testing of OPLS-DA **(D)**.

**FIGURE 6:**
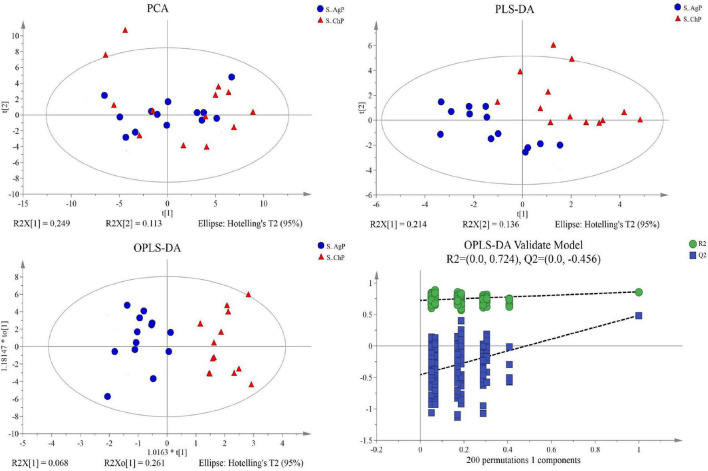
Dissimilarity in salivary metabolic signatures between AgP and ChP. PCA analysis **(A)**, PLS-DA analysis **(B)**, OPLS-DA analysis **(C)**, and response permutation testing of OPLS-DA **(D)**.

Multivariate analyses between different groups were performed in PCA, PLS-DA, and OPLS-DA. In the plot of PCA scores, we noted a trend of divergence in the saliva sample among the three groups. The PLS-DA model demonstrated satisfactory modeling and predictive abilities. The OPLS-DA model demonstrated a distinct separation between the metabolite profiles of these groups (Figures 4–6). The dissimilarity tests, including ANOSIM, Adonis, and MRPP, indicated significant differences in metabolites among the three groups (Table 3).

**TABLE 3:** Differences in metabolome among the three groups (*p* value).

	ANOSIM	Adonis	MRPP
S_AgP vs. S_H	0.024*	0.03*	0.023*
S_ChP vs. S_H	0.023*	0.018*	0.012*
S_AgP vs. S_ChP	0.047*	0.047*	0.04*

*p < 0.05.

**FIGURE 7:**
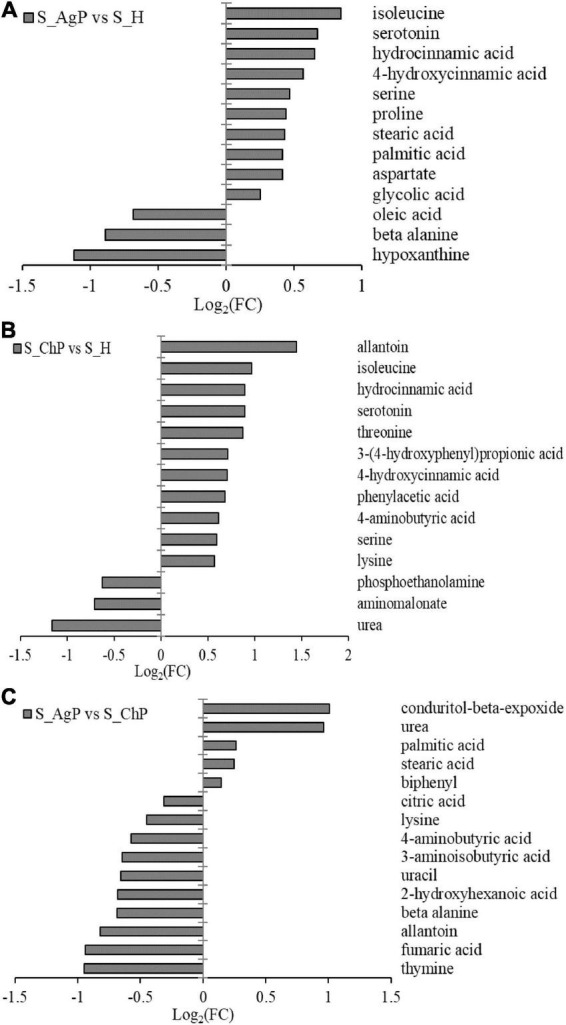
Significantly different metabolites between groups. Differential metabolites between AgP and PH. A positive value indicates a relatively higher concentration present in AgP, while a negative value means a relatively higher concentration in the PH **(A)**, based on differential metabolites between ChP and PH. A positive value indicates a relatively higher concentration present in ChP, while a negative value means a relatively higher concentration in the PH **(B)**, based on differential metabolites between AgP and ChP. A positive value indicates a relatively higher concentration present in AgP, while a negative value means a relatively higher concentration in the ChP **(C)**.

There were 13, 14, and 15 metabolites that were distributed differently between AgP vs. PH, ChP vs. PH, and AgP vs. ChP, respectively (Figure 7). Among these compounds, the levels of isoleucine, serotonin, hydrocinnamic acid, 4-hydroxycinnamic acid, serine, proline, stearic acid, palmitic acid, aspartate, and glycolic acid were significantly higher in AgP, while the levels of hypoxanthine, beta-alanine, and oleic acid were significantly higher in PH. Compared with PH, subjects with ChP are characterized by lower levels of urea, aminomalonate, and phosphoethanolamine, and higher levels of allantoin, isoleucine, hydrocinnamic acid, serotonin, threonine, 3-(4-hydroxyphenyl)propionic acid, 4-hydroxycinnamic acid, phenylacetic acid, 4-aminobutyric acid, serine, and lysine. Compared with ChP, the levels of biphenyl, stearic acid, palmitic acid, urea, and conduritol-beta-expoxide increased significantly in AgP, while the levels of thymine, fumaric acid, allantoin, beta-alanine, 2-hydroxyhexanoic acid, uracil, 3-aminoisobutyric acid, 4-aminobutyric acid, lysine, and citric acid were significantly decreased.

### Pathways and enrichment

Following the enrichment analysis of the significantly different metabolites, the key pathways with the highest correlation between metabolite differences were identified based on both raw p and impact values.

**FIGURE 8:**
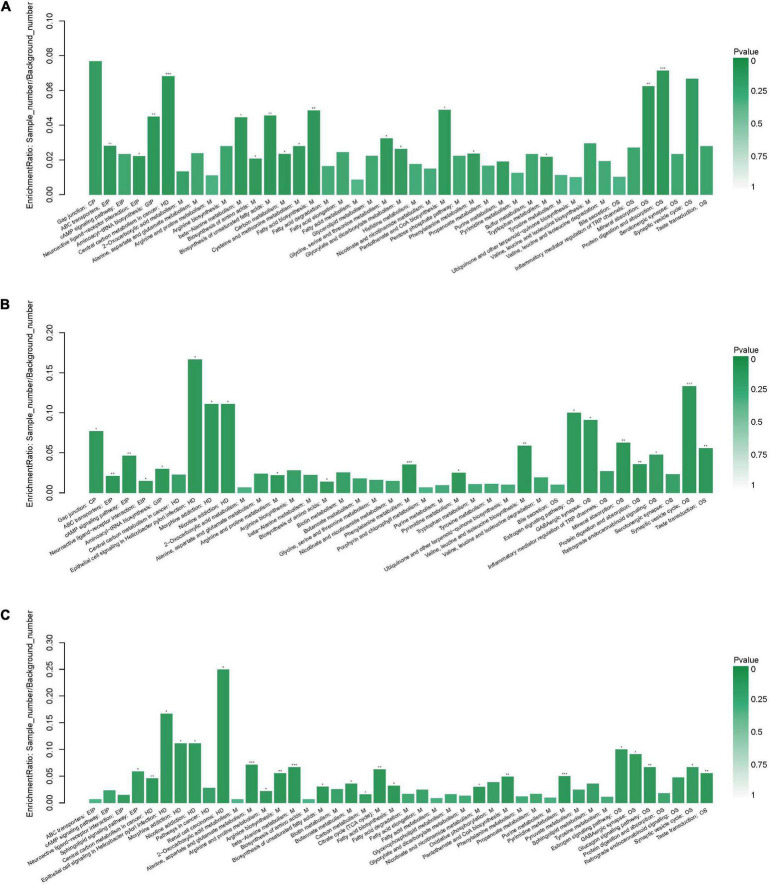
Functional enrichment analysis of differential metabolites between groups. Pathway analysis of salivary metabolites with significant difference between AgP and PH **(A)**, between ChP and PH **(B)**, and between AgP and ChP **(C)**. The height of the column represents the enrichment rate. *p < 0.05, **p < 0.01, ***p < 0.001.

When the AgP and PH groups were compared, several pathways containing more types of differential metabolites were detected, including protein digestion and absorption (including beta-alanine, proline, isoleucine, aspartate, and serine), biosynthesis of antibiotics (including serine, proline, isoleucine, glycolic acid, and aspartate), biosynthesis of amino acids (including serine, proline, isoleucine, and aspartate), aminoacyl-tRNA biosynthesis (including serine, proline, isoleucine, and aspartate), and fatty acid biosynthesis (including palmitic acid, stearic acid, and oleic acid) (Figure 8A).

When the ChP and PH groups were compared, several pathways containing more types of differential metabolites were detected, including aminoacyl-tRNA biosynthesis (including serine, isoleucine, lysine, and threonine), biosynthesis of amino acids (including serine, isoleucine, lysine, and threonine), and biosynthesis of valine, leucine, and isoleucine (including isoleucine and threonine) (Figure 8B).

When the ChP and AgP groups were compared, several pathways containing more types of differential metabolites were identified, including pyrimidine metabolism (including uracil, urea, thymine, and beta-alanine); alanine, aspartate, and glutamate metabolism (including citric acid, fumaric acid, and 4-aminobutyric acid); beta-alanine metabolism (including uracil, beta-alanine, and 4-aminobutyric acid); citrate cycle (including citric acid and fumaric acid); and arginine and proline metabolism (including urea and 4-aminobutyric acid) (Figure 8C).

### Association between microbial community and metabolome characteristics

Spearman correlation analysis was performed between the differential metabolites, the microbial diversity was analyzed at the phylum and genus levels, and the related network was constructed (| correlation r| > 0.25, *p* < 0.05).

**FIGURE 9:**
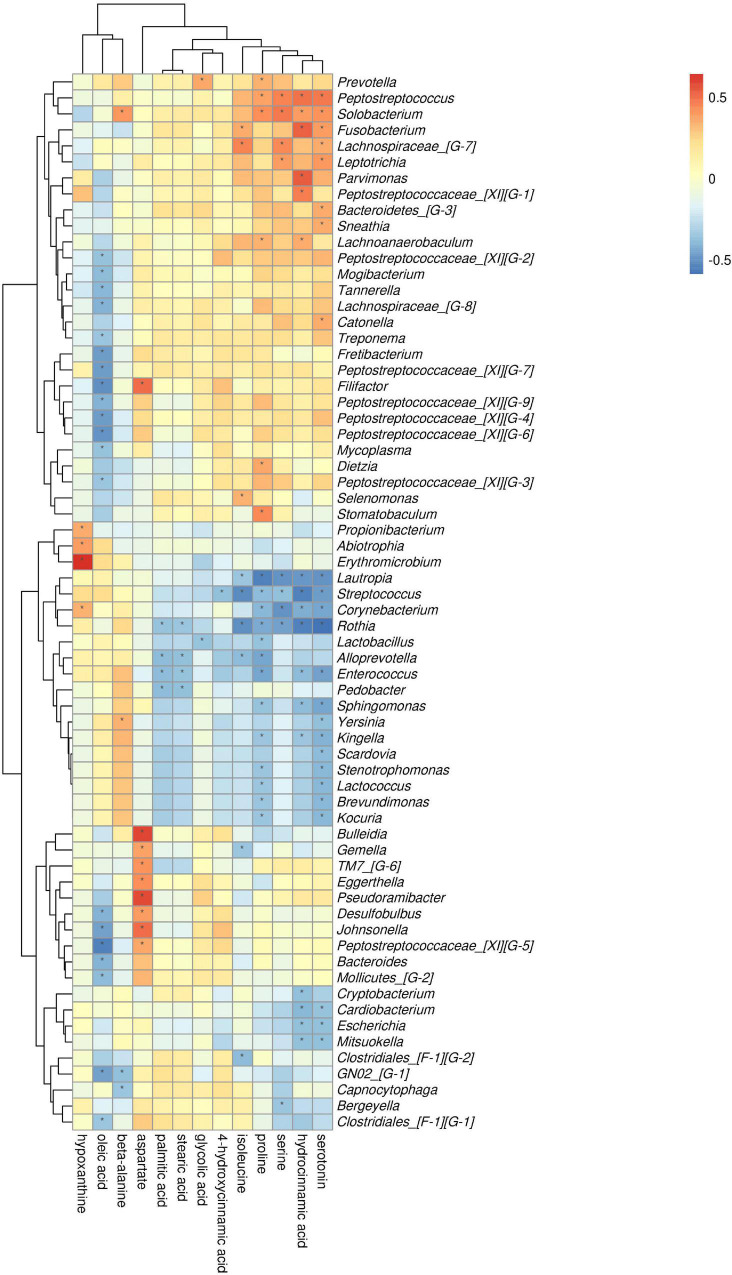
Association of microbial genera with differential metabolites in AgP and PH. Color intensity represents the magnitude of correlation. Red, positive correlations; blue, negative correlations. *Significant correlation between the genus and metabolite (*p* < 0.05).

At the phylum level, by comparing AgP and PH groups, we found that Actinobacteria and Fusobacteria were the most closely related to the metabolites (Supplementary Figure 2). At the genus level, *Rothia*, *Streptococcus*, *Corynebacterium*, *Enterococcus, Lautropia*, and *Solobacterium* were the genera with the most correlations with differential metabolites, which had significant correlations with 7, 6, 5, 5, 5, and 5 types of metabolites, respectively (Figure 9). Analysis from the perspective of metabolites showed that serotonin, oleic acid, proline, and hydrocinnamic acid had the most significant correlations with microorganisms, which were significantly correlated with 24, 20, 19, and 17 types of bacterial genera, respectively.

**FIGURE 10:**
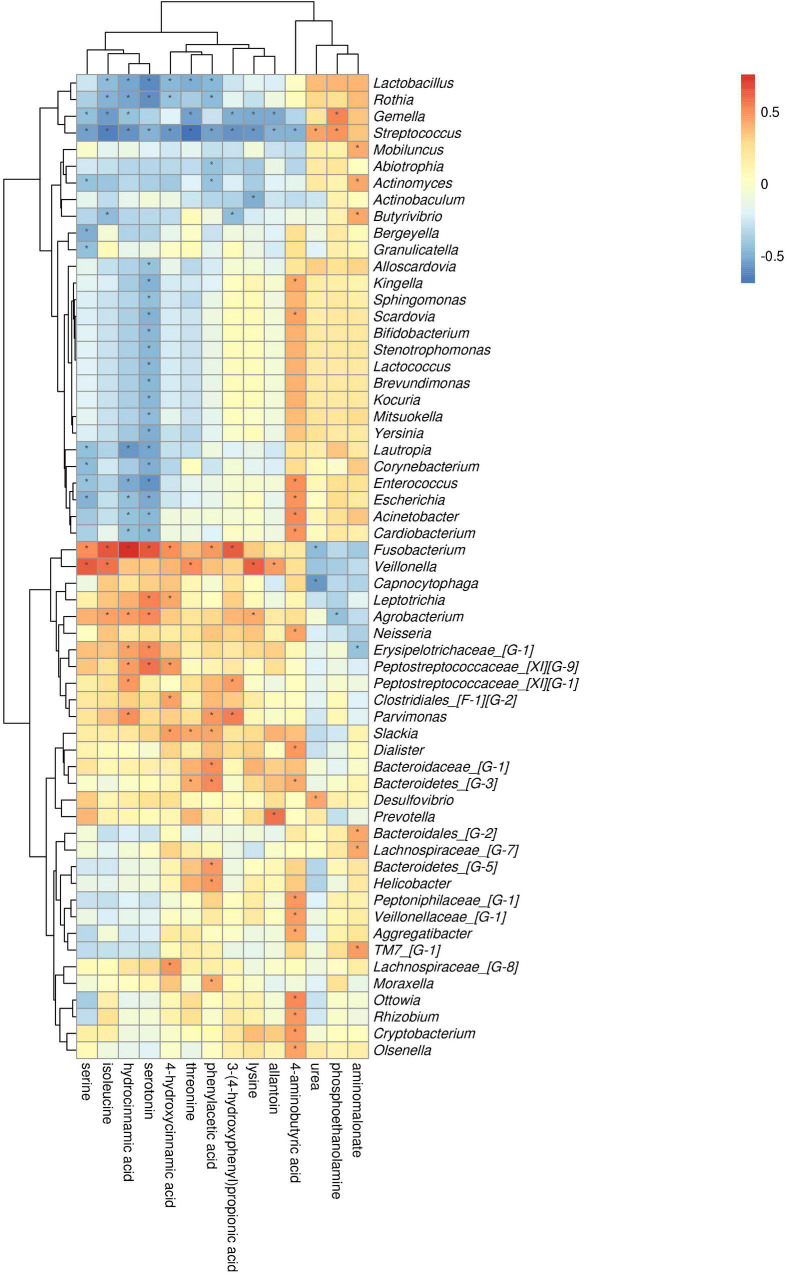
Association of microbial genera with differential metabolites in ChP and PH.

The comparison of ChP and PH groups revealed that Firmicutes, Actinobacteria, and Fusobacteria were most closely related to the metabolites (Supplementary Figure 3). At the genus level, *Streptococcus*, *Fusobacterium*, and *Gemella* were the genera with the most correlations with differential metabolites, which had significant correlations with 13, 8, and 8 types of metabolites, respectively (Figure 10). Lysine, aminobutyric acid, hydrocinnamic acid, and phenylacetic acid had the most significant correlations with microorganisms, which were significantly correlated with 25, 17, 15, and 13 types of bacterial genera, respectively.

**FIGURE 11:**
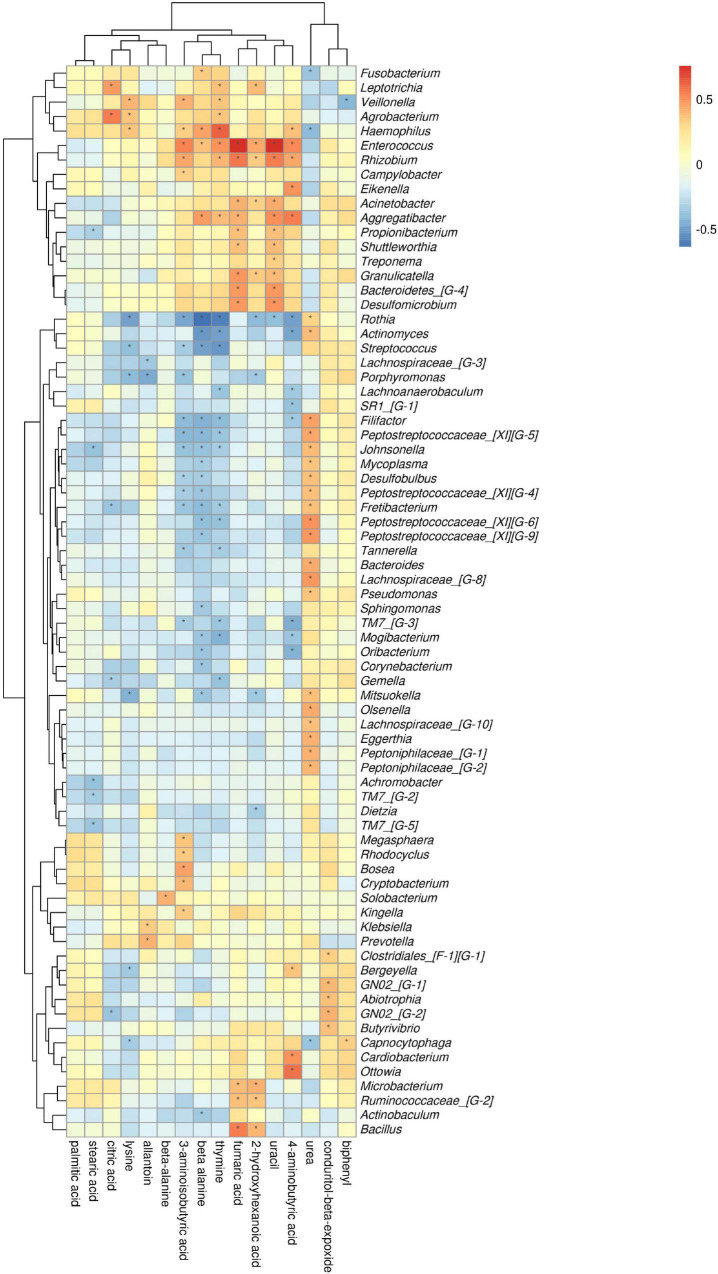
Association of microbial genera with differential metabolites in AgP and ChP.

The comparison of ChP and AgP groups showed that Actinobacteria, Synergistetes, Fusobacteria, and Proteobacteria were the most closely related to the metabolites (Supplementary Figure 4). At the genus level, *Rothia*, *Enterococcus*, *Rhizobium*, and *Haemophilus* are the genera with the most correlations with differential metabolites, which had significant correlations with 8, 7, 6, and 6 types of metabolites, respectively (Figure 11). Urea, beta-alanine, 3-aminoisobutyric acid, and thymine had the most significant correlations with microorganisms, which were significantly correlated with 23, 22, 21, and 20 types of bacterial genera, respectively.

## Discussion

Periodontitis is a biofilm-associated inflammatory condition that is associated with the activation of the host inflammatory response to bacterial challenge, which mainly includes chronic periodontitis and aggressive periodontitis. This pilot study provided a comprehensive and comparative analysis of the microbiome and metabolome of salivary samples from individuals with AgP, ChP, and PH.

As the present study was conducted between January 2017 and August 2017, the included patients were diagnosed with AgP or ChP, according to the 1999 classification system. When the new classification system was presented in 2018, the authors reclassified the groups according to the new classification system and revealed that all patients fully met the criteria for generalized Stage III or IV and Grade C periodontitis features. However, the terms “AgP” and “ChP” were retained, as the results of the present study were also compared with the previous studies on AgP and ChP.

Saliva is the most easily accessible and readily obtained biofluid, which is enriched with microbiota from the totality of the oral cavity, including the subgingival region where periodontitis occurs (Acharya et al., 2017; Krishnan et al., 2017). Despite the progressive development of high-throughput sequencing techniques, there are still a limited number of studies investigating the salivary microbial composition of periodontitis.

Our results reported that the alpha diversity of subjects with periodontitis was greater than that observed in healthy individuals, which is in agreement with the previous studies (Ko et al., 2020; Sun et al., 2020). In line with a previous study, there were significant differences in β diversity values between periodontitis and control, while there was no significant difference in the salivary microbial data between AgP and ChP (Damgaard et al., 2019). Nibali et al. (2021) also indicated that limited microbial differences existed in the subgingival microbial communities between AgP and ChP. Salivary bacteria have been characterized in healthy individuals, and the predominant phyla were found to be Firmicutes, Bacteroidetes, Proteobacteria, Fusobacteria, and Actinobacteria (Segata et al., 2012). In this study, the phyla Spirochaetes and Synergistetes and the genera *Treponema*, *Peptococcus*, *Catonella*, *Desulfobulbus*, *Peptostreptococcaceae_[XI] ([G-2]*, *[G-3] [G-4]*, *[G-6]*, and *[G-9])*, *Bacteroidetes_[G-5]*, *TM7_[G-5]*, *Dialister*, *Eikenella*, *Fretibacterium*, and *Filifactor* were present at higher levels in patients with periodontitis than in healthy participants. The association between these taxa in saliva and periodontitis has been well demonstrated (Lundmark et al., 2019; Ko et al., 2020; Sun et al., 2020). In addition, these genera have also previously been associated with periodontitis in subgingival plaque samples. *Treponema*, *Desulfobulbus*, *Catonella*, *Bacteroides*, *Peptostreptococcus*, and *Eikenella* have been reported as periodontitis biomarkers (Cai et al., 2021). *Filifactor*, *Fretibacterium*, *Peptostreptococcaceae_[XI][G-5]*, *Peptostreptococcaceae_[XI][G-6]*, *Peptostreptococcus*, and *Treponema* were significantly higher in the AgP group (Shi et al., 2020).

Metabolomics is a newly emerging technique that has been increasingly used to discover biomarkers for the diagnosis and prognosis of periodontitis. However, “chronic” and “aggressive” are now grouped as “Periodontitis” according to the current classification system (Papapanou et al., 2018). Significant differences in the composition of salivary metabolites among the three groups were found in the present study. In previous studies, AgP and ChP metabolomic profiles were not unequivocally discriminated through the NMR-based spectroscopic analysis of saliva (Rzeznik et al., 2017; Romano et al., 2018). The reason for this inconsistency might be based on different testing methods. The metabolites could be released due to bacterial metabolism or host-induced inflammatory processes, which suggested host–bacteria interactions in AgP may be different when compared with ChP. AgP displays an inadequate host response to periodontopathogenic bacteria, which can be attributed to increased expression of a wide variety of immunological and genetic risk factors (Takahashi et al., 2001; Yoshida et al., 2021).

Significantly higher levels of serine, serotonin, 4-hydroxycinnamic acid, hydrocinnamic acid, and isoleucine were identified in both the AgP and ChP groups when compared with the healthy controls in this study. The reports of previous studies on metabolite changes of saliva due to periodontitis match our findings, including isoleucine and proline (Romano et al., 2018; Citterio et al., 2020), threonine (Sugimoto et al., 2010; Rzeznik et al., 2017), and 4-aminobutyric acid (Sakanaka et al., 2017). Isoleucine reflects the host immune response to the oral microbiome and has already been associated with active periodontal disease (Zhang et al., 2017). The higher level of proline in AgP could be explained by the up-regulation of protease activity found in periodontitis (Wishart, 2008). The biochemical pathways that are increased in patients with periodontitis compared with healthy subjects include protein digestion and absorption, biosynthesis of amino acids, aminoacyl-tRNA biosynthesis, and glycine, serine, and threonine metabolism. Aminoacyl-tRNA determines how the genetic code is translated into amino acids. The biochemical pathways that are significantly different between the ChP and AgP groups include pyrimidine metabolism, beta-alanine metabolism, alanine, aspartate and glutamate metabolism, citrate cycle, and arginine and proline metabolism. Pyrimidine metabolism has been reported to be associated with AgP (Pei et al., 2020; Shi et al., 2020). Arginine and proline metabolism has been demonstrated to be involved in periodontitis mechanism (Kouznetsova et al., 2021). For example, degradation of arginine and other amino acids occurs in the periodontal pockets by asaccharolytic anaerobic Gram-positive rods (Uematsu et al., 2003). Arginine metabolism to nitric oxide also plays a role in periodontitis (Ozer et al., 2011).

To our knowledge, we reported the combined interactions between salivary microbiota and metabolites for the first time. In the association analysis of differential metabolites and microorganisms, the results showed that genera with the same change in trend between groups tended to have a similar correlation with some certain metabolites, either positively or negatively. The abundance of *Streptococcus* was significantly higher in PH than in AgP and ChP, which was positively correlated with the majority of healthy control-enriched metabolites and negatively correlated with the majority of periodontitis-enriched metabolites. Hydrocinnamic acid had significant related genera with 17 types in AgP and 15 types in ChP, respectively. The differential metabolites between the ChP and AgP groups, such as urea, beta-alanine, 3-aminoisobutyric acid, and thymine, had the most significant correlations with genera. However, the specific mechanism remains to be further explored and confirmed.

In conclusion, distinct differences were observed in salivary microbiome and metabolomics between periodontitis and healthy control. Striking differences were found in the composition of salivary metabolites between the AgP and ChP groups. The integration of microbial data and metabolomic data may help us understand the potential mechanism of periodontitis and offer potential biomarkers. A larger sample size is needed to validate the findings. Another limitation of our study is the differences in age distribution among the three groups that might potentially affect the results. In addition, it has already been demonstrated that salivary metabolites are not affected by gender, body mass index, and dietary intake (Walsh et al., 2006; Bertram et al., 2009). In addition, Kim et al. (2021) indicated that age differences did not affect the metabolite profile of saliva. A further longitudinal study in a larger cohort study, including dynamic changes in the salivary microbiome and metabolome after the initial treatment, would provide a better understanding of periodontal host–microbial interactions.

## Data availability statement

The datasets presented in this study can be found in online repositories. The names of the repository/repositories and accession number(s) can be found in the article/Supplementary material.

## Ethics statement

The studies involving human participants were reviewed and approved by Ethics Committee of the Peking University Health Science Center. The patients/participants provided their written informed consent to participate in this study.

## Author contributions

WH and XW: conception and design. YW, MS, FS, and WJ: recruitment of patients and collection of oral samples. MS, YW, and YN: analysis and interpretation of data. YW and MS: manuscript preparation. MS, YW, YN, CW, and WH: manuscript revision. All authors contributed to the article and approved the submitted version.

## Footnotes

1
https://cloud.majorbio.com/

## Supplementary material

Supplementary Figure 1 Rarefaction curves.

Supplementary Figure 2 Correlations between salivary plaque bacteria (phylum level) with differential metabolites between AgP and PH. Each row in the graph represents a phylum, each column represents a metabolite, and each lattice represents a correlation coefficient between a component and a metabolite. Red represents a positive correlation, while blue represents a negative correlation. *Significant correlation between the phyla and metabolites (*p* < 0.05).

Supplementary Figure 3 Correlations between salivary plaque bacteria (phylum level) with differential metabolites between ChP and PH.

Supplementary Figure 4 Correlations between salivary plaque bacteria (phylum level) with differential metabolites between AgP and ChP.
