# Supplementary figures and images for: Integrated analysis of the salivary microbiome and metabolome in chronic and aggressive periodontitis: A pilot study
Source: Front Microbiol. 2022 Sep 26;13:959416. doi: 10.3389/fmicb.2022.959416 (PMC9549375; doi:10.3389/fmicb.2022.959416)

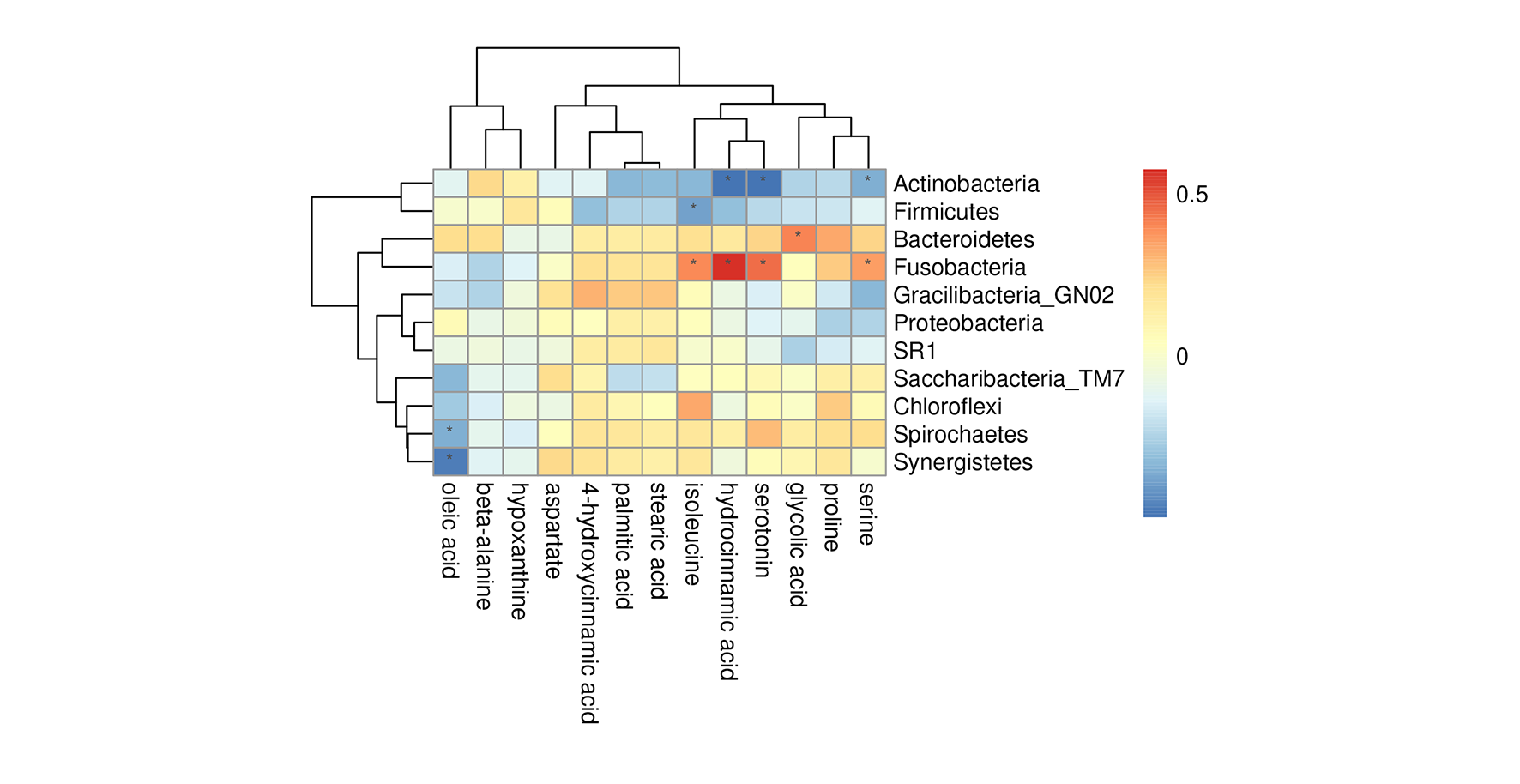

Supplement: Supplementary Figure 2 — Correlations between salivary plaque bacteria (phylum level) with differential metabolites between AgP and PH. Each row in the graph represents a phylum, each column represents a metabolite, and each lattice represents a correlation coefficient between a component and a metabolite. Red represents a positive correlation, while blue represents a negative correlation. *Significant correlation between the phyla and metabolites (p < 0.05). [file Image_2.TIF]

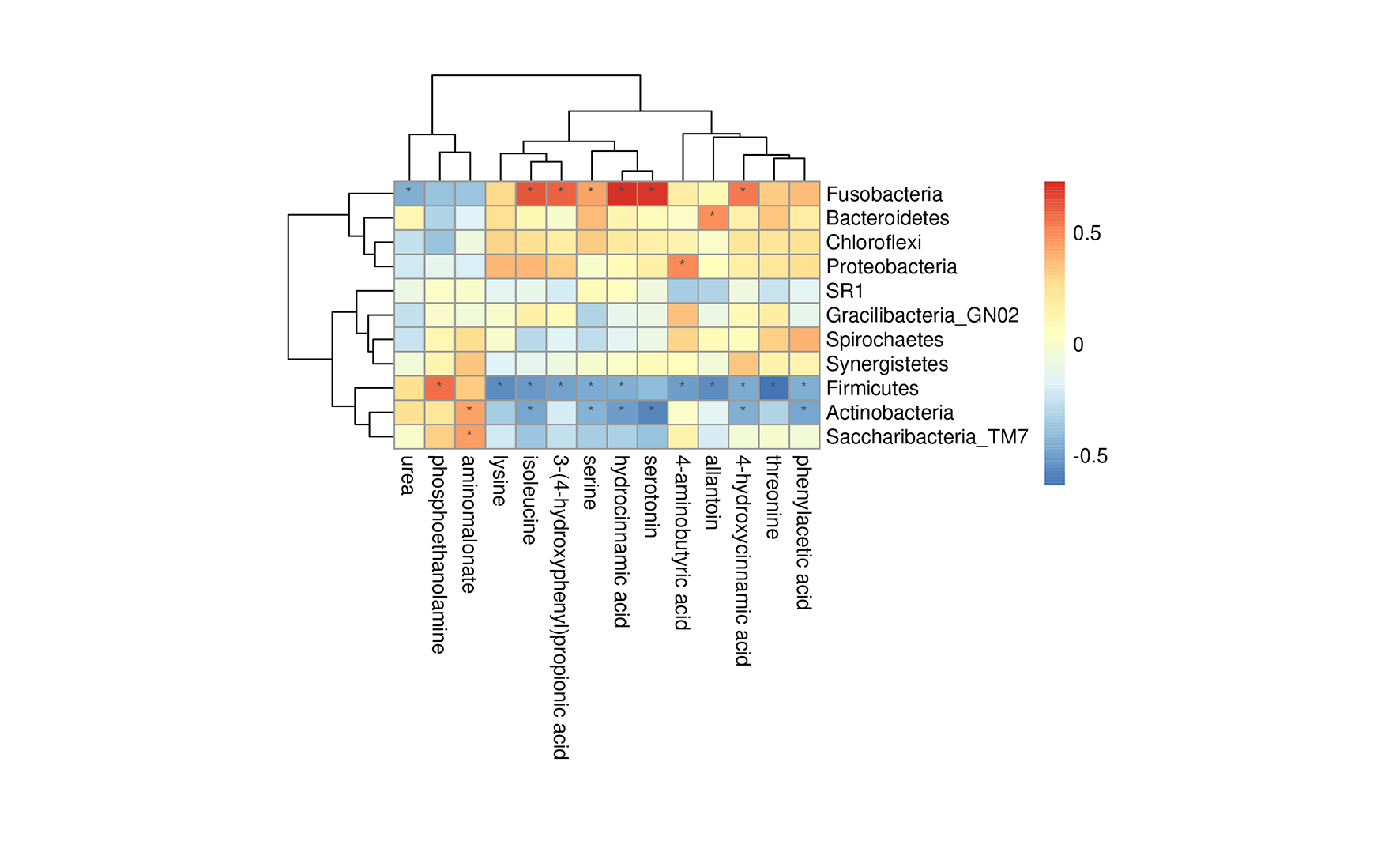

Supplement: Supplementary Figure 3 — Correlations between salivary plaque bacteria (phylum level) with differential metabolites between ChP and PH. [file Image_3.TIF]

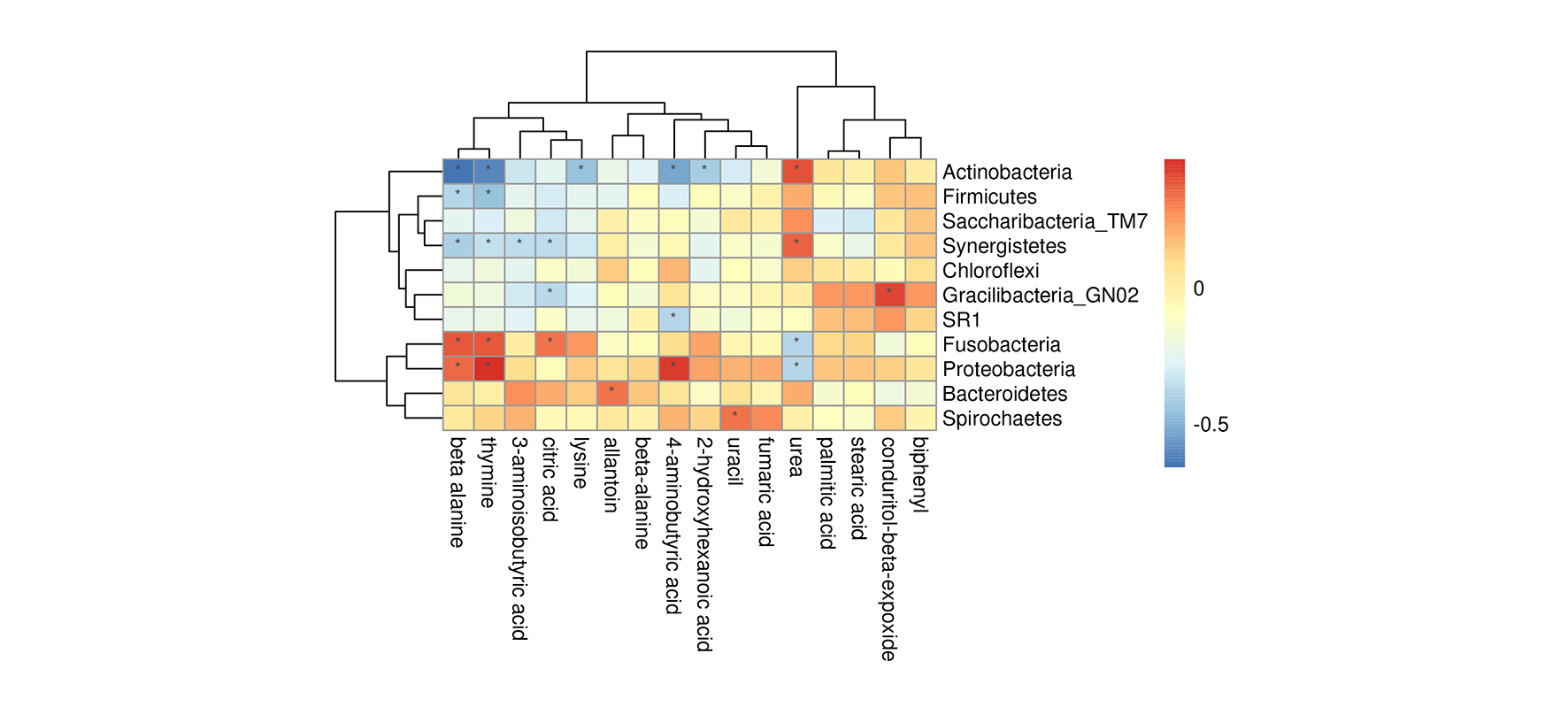

Supplement: Supplementary Figure 4 — Correlations between salivary plaque bacteria (phylum level) with differential metabolites between AgP and ChP. [file Image_4.TIF]
