# Supplementary material for: Integrated analysis of the salivary microbiome and metabolome in chronic and aggressive periodontitis: A pilot study
Source: Front Microbiol. 2022 Sep 26;13:959416. doi: 10.3389/fmicb.2022.959416 (PMC9549375; doi:10.3389/fmicb.2022.959416)
Supplement: Supplementary file 5 [file Data_Sheet_1.docx]

Supplementary Table 1 Differential genera in saliva samples from patients with aggressive periodontitis compared to healthy controls

| genus | S_AgP-Mean(%) | S_H-Mean(%) | *p* value |
| --- | --- | --- | --- |
| *Actinobaculum* | 0.08894 | 0.03175 | 0.044020 |
| *Bacteroidales_[G-2]* | 0.03 | 0.008699 | 0.028300 |
| *Bacteroides* | 0.01005 | 0.000435 | 0.007980 |
| *Bacteroidetes_[G-3]* | 0.0237 | 0.00174 | 0.000787 |
| *Bacteroidetes_[G-5]* | 0.0294 | 0.000435 | 0.000165 |
| *Catonella* | 0.2692 | 0.04784 | 0.000202 |
| *Desulfobulbus* | 0.01065 | 0 | 0.019110 |
| *Dialister* | 0.08564 | 0.02523 | 0.010780 |
| *Eggerthia* | 0.008849 | 0 | 0.006019 |
| *Eikenella* | 0.06989 | 0.03871 | 0.011920 |
| *Erysipelotrichaceae_[G-1]* | 0.006149 | 0 | 0.009020 |
| *Filifactor* | 0.4543 | 0.01218 | 0.000012 |
| *Fretibacterium* | 0.05504 | 0.002175 | 0.000152 |
| *Fusobacterium* | 7.671 | 4.538 | 0.029930 |
| *GN02_[G-2]* | 0.4117 | 0.02131 | 0.017480 |
| *Helicobacter* | 0.0027 | 0 | 0.037650 |
| *Johnsonella* | 0.0117 | 0 | 0.006171 |
| *Lachnospiraceae_[G-8]* | 0.07259 | 0.005654 | 0.000673 |
| *Leptotrichia* | 2.65 | 1.314 | 0.002370 |
| *Mollicutes_[G-1]* | 0.01485 | 0 | 0.013330 |
| *Mollicutes_[G-2]* | 0.00255 | 0 | 0.037450 |
| *Mycoplasma* | 0.04545 | 0.003045 | 0.004337 |
| *Olsenella* | 0.07139 | 0.01479 | 0.011920 |
| *Parvimonas* | 0.9237 | 0.9482 | 0.006887 |
| *Peptococcus* | 0.2047 | 0.04393 | 0.000865 |
| *Peptoniphilaceae_[G-1]* | 0.0174 | 0.001305 | 0.012830 |
| *Peptostreptococcaceae_[XI][G-2]* | 0.0255 | 0.006089 | 0.024810 |
| *Peptostreptococcaceae_[XI][G-3]* | 0.005999 | 0 | 0.037980 |
| *Peptostreptococcaceae_[XI][G-4]* | 0.07979 | 0.00174 | 0.000017 |
| *Peptostreptococcaceae_[XI][G-5]* | 0.2623 | 0.02871 | 0.000912 |
| *Peptostreptococcaceae_[XI][G-6]* | 0.1368 | 0.003045 | 0.000146 |
| *Peptostreptococcaceae_[XI][G-9]* | 0.2019 | 0.006524 | 0.000024 |
| *Peptostreptococcus* | 3.433 | 2.692 | 0.041110 |
| *Propionibacterium* | 0.05549 | 0.04437 | 0.014270 |
| *Slackia* | 0.008999 | 0.000435 | 0.026130 |
| *Solobacterium* | 0.4197 | 0.2336 | 0.044390 |
| *Streptococcus* | 26.19 | 36.54 | 0.023360 |
| *Tannerella* | 0.08294 | 0.02784 | 0.007157 |
| *TM7_[G-2]* | 0.0036 | 0 | 0.018700 |
| *TM7_[G-4]* | 0.03345 | 0.00087 | 0.008305 |
| *TM7_[G-5]* | 0.02655 | 0.01392 | 0.015660 |
| *Treponema* | 0.625 | 0.03567 | 0.000025 |
| *Veillonellaceae_[G-1]* | 0.0345 | 0.01566 | 0.004066 |

Supplementary Table 2 Differential genera in saliva samples from patients with chronic periodontitis compared to healthy controls

| genus | S_ChP-Mean(%) | S_H-Mean(%) | *p* value |
| --- | --- | --- | --- |
| *Peptostreptococcaceae_[XI][G-9]* | 0.07471 | 0.006524 | 0.00038 |
| *Filifactor* | 0.264 | 0.01218 | 0.000385 |
| *Peptostreptococcaceae_[XI][G-4]* | 0.04145 | 0.00174 | 0.000403 |
| *Catonella* | 0.2134 | 0.04784 | 0.002788 |
| *Erysipelotrichaceae_[G-1]* | 0.006908 | 0 | 0.003971 |
| *Bacteroidetes_[G-5]* | 0.03045 | 0.000435 | 0.004863 |
| *Treponema* | 0.7486 | 0.03567 | 0.005153 |
| *Dialister* | 0.1016 | 0.02523 | 0.007774 |
| *Fretibacterium* | 0.03889 | 0.002175 | 0.009509 |
| *Slackia* | 0.006141 | 0.000435 | 0.01158 |
| *Leptotrichia* | 3.841 | 1.314 | 0.01294 |
| *Lachnospiraceae_[G-8]* | 0.03556 | 0.005654 | 0.01357 |
| *Mollicutes_[G-1]* | 0.008443 | 0 | 0.01391 |
| *Peptostreptococcaceae_[XI][G-3]* | 0.004861 | 0 | 0.01393 |
| *Johnsonella* | 0.01151 | 0 | 0.014 |
| *Veillonellaceae_[G-1]* | 0.04324 | 0.01566 | 0.01797 |
| *Eikenella* | 0.06089 | 0.03871 | 0.0192 |
| *Peptostreptococcaceae_[XI][G-2]* | 0.03249 | 0.006089 | 0.02334 |
| *TM7_[G-5]* | 0.03608 | 0.01392 | 0.02381 |
| *Desulfobulbus* | 0.004094 | 0 | 0.02442 |
| *Lactobacillus* | 0.000256 | 0.00261 | 0.03113 |
| *Streptococcus* | 22.57 | 36.54 | 0.03285 |
| *Peptococcus* | 0.2136 | 0.04393 | 0.03878 |
| *Bergeyella* | 0.1251 | 0.1605 | 0.03926 |
| *Parvimonas* | 1.221 | 0.9482 | 0.03944 |
| *Agrobacterium* | 0.001535 | 0 | 0.03983 |
| *Pseudoramibacter* | 0.003326 | 0 | 0.04129 |
| *Eggerthia* | 0.005117 | 0 | 0.04164 |
| *Bulleidia* | 0.00742 | 0.002175 | 0.04207 |
| *Peptostreptococcaceae_[XI][G-6]* | 0.05578 | 0.003045 | 0.04521 |
| *Solobacterium* | 0.5186 | 0.2336 | 0.04734 |

Supplementary Table 3 Differential genera in saliva samples from patients with aggressive periodontitis compared to patients with chronic periodontitis

| genus | S_AgP-Mean(%) | S_ChP-Mean(%) | *p* value |
| --- | --- | --- | --- |
| *Agrobacterium* | 0.00015 | 0.001535 | 0.004338 |
| *Mobiluncus* | 0.0039 | 0.000256 | 0.02801 |
| *GN02_[G-1]* | 0.007049 | 0.002559 | 0.02932 |
| *GN02_[G-2]* | 0.4117 | 0.06089 | 0.04454 |
| *Peptostreptococcaceae_[XI][G-6]* | 0.1368 | 0.05578 | 0.04771 |
